# Space-time clustering of recently-diagnosed tuberculosis and impact of ART scale-up: Evidence from an HIV hyper-endemic rural South African population

**DOI:** 10.1038/s41598-019-46455-7

**Published:** 2019-07-24

**Authors:** Andrew Tomita, Catherine M. Smith, Richard J. Lessells, Alexander Pym, Alison D. Grant, Tulio de Oliveira, Frank Tanser

**Affiliations:** 1 Africa Health Research Institute, KwaZulu-Natal, South Africa; 2 KwaZulu-Natal Research Innovation and Sequencing (KRISP), College of Health Sciences, University of KwaZulu-Natal, Durban, South Africa; 3 Centre for Rural Health, School of Nursing and Public Health, University of KwaZulu-Natal, Durban, South Africa; 4 Institute of Health Informatics, University College London, London, UK; 5 School of Nursing and Public Health, University of KwaZulu-Natal, Durban, South Africa; 6 Research Department of Infection & Population Health, University College London, London, UK; 7 TB Centre, London School of Hygiene & Tropical Medicine, London, UK; 8 School of Public Health, University of the Witwatersrand, Johannesburg, South Africa; 9 Centre for the AIDS Programme of Research in South Africa (CAPRISA), Durban, South Africa; 10 Department of Global Health, University of Washington, Seattle, USA

**Keywords:** Infectious diseases, Epidemiology

## Abstract

In HIV hyperendemic sub-Saharan African communities, particularly in southern Africa, the likelihood of achieving the Sustainable Development Goal of ending the tuberculosis (TB) epidemic by 2030 is low, due to lack of cost-effective and practical interventions in population settings. We used one of Africa’s largest population-based prospective cohorts from rural KwaZulu-Natal Province, South Africa, to measure the spatial variations in the prevalence of recently-diagnosed TB disease, and to quantify the impact of community coverage of antiretroviral therapy (ART) on recently-diagnosed TB disease. We collected data on TB disease episodes from a population-based sample of 41,812 adult individuals between 2009 and 2015. Spatial clusters (‘hotspots’) of recently-diagnosed TB were identified using a space-time scan statistic. Multilevel logistic regression models were fitted to investigate the relationship between community ART coverage and recently-diagnosed TB. Spatial clusters of recently-diagnosed TB were identified in a region characterized by a high prevalence of HIV and population movement. Every percentage increase in ART coverage was associated with a 2% decrease in the odds of recently-diagnosed TB (aOR = 0.98, 95% CI:0.97–0.99). We identified for the first time the clear occurrence of recently-diagnosed TB hotspots, and quantified potential benefit of increased community ART coverage in lowering tuberculosis, highlighting the need to prioritize the expansion of such effective population interventions targeting high-risk areas.

## Introduction

Tuberculosis (TB) is the leading cause of death from infectious disease in the world^1^, with epidemics being spatially heterogeneous, as indicated by evidence of geographic clustering at different resolution levels^2,3^. Given the lack of consistent evidence for scalable community-wide approaches to control TB^4^, particularly in resource-limited regions grappling with the realities of an unrelenting HIV endemic, there is considerable interest in developing sustainable, targeted interventions in a precision public health approach^5^. TB accounts for the largest percentage of total mortality in 2016 (6.5%) in South Africa^6^ and is the fifth leading cause of years of life lost^7^ and disability-adjusted life-years in the country^8^, according to the Global Burden of Disease Study 2015. In sub-Saharan Africa (SSA), particularly southern Africa, as HIV is the major driver of the TB epidemic^9–11^, this also raises questions around how geographically targeted HIV interventions could influence TB epidemiology.

The rapid expansion of access to antiretroviral therapy (ART) in high HIV prevalence countries has had a profound impact on population health, having improved life expectancy and contributed to reductions in new infections^12,13^. ART is strongly associated with a reduced risk of TB disease in HIV-positive individuals^30^, and some evidence suggests that increasing ART coverage has had a significant impact on the epidemiology of TB in countries with an associated high prevalence of HIV. National-level analyses in Kenya, Malawi, South Africa and Zimbabwe have demonstrated reductions in TB notifications coincident with increasing ART coverage^14–20^. These findings have been consolidated in a recent systematic review, which reported consistent declines in TB notification rates in most SSA countries between 2010 and 2015^21^.

At a more local level, studies from Cape Town, South Africa, and rural Malawi have demonstrated an association between increasing ART coverage and declining TB case notification rate^22–24^. One community study from Cape Town also showed a significant decline in the prevalence of undiagnosed TB in the early phase of ART roll-out as coverage expanded from 5% to 20%^25^. However, none of these studies had access to individual-level HIV and ART data, with the specific effect of ART on TB epidemiology at a population level therefore not being well understood.

Mathematical models have suggested that although expanded ART coverage will lead to an initial decline in TB incidence, this might stabilize or be followed by a rebound if not accompanied by other prevention strategies^26–29^. According to a systematic review and meta-analysis^30^, ART may be associated with the reduced risk of HIV-associated TB disease in HIV-positive individuals due to a lowering of their viral load and improvements in their immune system function. On one hand, while ART reduces new HIV infections, on the other hand, the marked decline in HIV-associated mortality has led to rising HIV prevalence and an increase in the number of life-years at risk of TB. Furthermore, it is plausible that the infectiousness of HIV-positive people with TB might increase with CD4+ T-cell recovery on ART, although the available data do not yet support this^31,32^. Most importantly, even in high HIV prevalence settings, HIV-positive people can be regarded as only making a relatively small contribution to TB transmission^33^, although this remains uncertain as ART coverage expands.

There are substantial geographical variations in the TB notification rates in South Africa, which do not correlate clearly with HIV prevalence at district level^17,34^. While this can be partially attributed to differences in TB detection rates, there is a need to better understand the factors that influence the spatial heterogenity of TB. This understanding and the population-level associations between HIV, ART coverage and TB, might provide important insight that could guide precision public health approaches to ending TB in high HIV prevalence communities. In this paper, we use data from one of the world’s largest longitudinal population health surveillance systems to characterize the spatial distribution of TB in a high HIV prevalence rural South African community and quantify the impact of community ART coverage scale-up on recently-diagnosed TB disease.

## Methods

### Study setting

Data from the Africa Health Research Institute’s (AHRI) demographic surveillance system were used to identify space-time clustering of recently-diagnosed TB (i.e. active TB), and to investigate the association between community-level ART coverage and TB. The AHRI demographic surveillance was launched in 2000 and is an extensive rural surveillance system that was primarily designed to collect longitudinal health and demographic data to monitor the severe HIV epidemic in a rural area of KwaZulu-Natal (KZN) Province, South Africa. The surveillance area of approximately 430 km^2^ is situated in the southern part of the uMkhanyakude District in northern KZN and consisted of approximately 87,000 inhabitants. The area, which is predominantly rural and populated mainly by isiZulu-speaking people, is characterized by a large variation in population densities (20–3,000 people/km^2^). The AHRI surveillance area is characterized by a persistently high HIV burden, with one report estimating the HIV incidence to be as high as 6.6 per 100 person-years in young women^13^. The TB case notification rate in the Hlabisa Health sub-District peaked at 1773 per 100,000 in 2008 and declined to 756 per 100,000 in 2014, with approximately three-quarters of the TB cases being HIV associated over this period. Between 2000 and 2011, HIV and TB were responsible for approximately half (49.2%) of all deaths in this community^35,36^.

### Data sources

The data consists of annually-collected information on individual-level health and socio-demographic factors (e.g. age, gender, marital status, HIV/TB status), as well as their living arrangement (e.g. geolocation, household assets/income). The data on individual HIV status was obtained from periodic HIV sero-surveys using the dried blood spot (DBS) sample method, based on two parallel tests using HIV-1/HIV-2 ELISA (Vironostika® HIV-1 Microelisa System; Biomérieux, Durham, NC, USA and Wellcozyme HIV 1 + 2 GACELISA; Murex Diagnostics Benelux B.V., Breukelen, the Netherlands). ART data for the study was obtained from AHRI’s ART Evaluation and Monitoring System (ARTemis). This system derives its data from the Hlabisa HIV Treatment and Care Programme, a KZN Department of Health initiative that is responsible for HIV treatment (i.e. ART) in the study area^37,38^. The individual-level ART data from ARTemis was subsequently link to the AHRI demographic surveillance data containing individual-level health and socio-demographic factors. A detailed description of the AHRI demographic surveillance system and ARTemis are published elsewhere^39^. Our eligible sample included adult individuals aged 15 years and above who participated in the individual general health questionnaire at least once anytime between 2009 and 2015. The household coordinates were obtained using global positioning systems and mapped using geographic information systems (GIS). The University of KwaZulu-Natal Biomedical Research Ethics Committee approved the implementation of the AHRI demographic surveillance, and informed consent was obtained from all participants, conforming to the South African Good Clinical Practice Guidelines and the Department of Health Ethics Guidelines. All methods were performed in accordance with the relevant guidelines and regulations.

### Outcome

The main individual-level outcome was TB, based on self-report, and recently-diagnosed within the previous 12 months from 2009–2015. The data were obtained from the annual general health questionnaire module from the AHRI demographic surveillance.

### Community-level HIV prevalence

The main study predictors were community-level HIV prevalence and community-level ART coverage for 2009–2012, for which individual ART data were available in our population health surveillance area. Community-level HIV prevalence was constructed using a geospatial grid-based technique using a GIS. This estimate was computed by means of a moving two-dimensional Gaussian kernel of 3 km search radius, a method consistent with our previous study^40^. The kernel moves systematically across the grid of cells and measures the spatial variation in HIV prevalence across each cell at household level, to which the positive cases have been linked and mapped. The resulting HIV prevalence and geographic distribution of the total eligible population were used to calculate the number of HIV-negative individuals in the area surrounding each cell on the grid. The mean HIV prevalence across communities in our surveillance area was between 22.63% and 24.85% during the observation years of 2009–2012.

### Community-level ART coverage

Community-level ART coverage was operationalized based on the methods from previously published work on community-level ART and HIV acquisition^13^. ART coverage, defined as the proportion of all HIV + individuals receiving ART, was calculated for each community within the surveillance area. A standard two-dimensional Gaussian kernel of a 3 km radius methodology was utilized, based on our previous work^41^, to produce a robust community-level estimate of ART coverage. ART was introduced to KZN in 2004. By 2009, the mean coverage across communities in the surveillance area was less than a third (30.19%). Coverage in ART, due to change in the treatment eligibility threshold, experienced accelerated expansion in the surveillance area in 2010^13^. The mean ART coverage increased to approximately half (51.91%) in 2012.

### Analyses

The data analysis consisted of two phases, with phase 1 identifying the spatial distribution and clustering of recently-diagnosed TB (**labeled ‘TB hotspot communities’ hereafter**), and phase 2 evaluating the association between community ART coverage and recently-diagnosed TB (**labeled ‘TB’ hereafter**). TB hotspot communities were defined as clusters of excess TB and identified using the Kulldorff space-time scan statistic, as described previously^41–43^. Scan statistics compared the observed number of TB cases within spatial windows of varying sizes with those that would be expected in the numbers of cases inside and outside the window. The prevalence of recently-diagnosed TB was summed for all individuals ≥15 years of age in each homestead and mapped to an accuracy of <2 m. The Kulldorff space-time scan statistic was then applied to identify clusters of TB, which were then compared to the general population under the Poisson distribution using R (spatstat and rsatscan packages^44,45^) and the SaTScan software^46^ (version 9.1). Clusters of TB (p < 0.05) were plotted on smoothed maps of the prevalence of TB in the area, generated using kernel density estimation and a standard Gaussian kernel. Following the identification of TB space-time clustering, multilevel mixed-effects logistic regressions were used to analyze the association between community-level ART coverage and TB using STATA 15^47^, and involved three levels of visits or repeated measures nested within in each person, individual and community.

The association between community ART coverage and recently-diagnosed TB was evaluated using five separate three-level random-intercept models that were sequentially fitted, with Model 0 fitting a null model without any explanatory variables. Model 1 provided the effect of individual-level predictors on TB without individual HIV/ART status, household and community predictors. Model 2 considered all the explanatory variables at individual-, household, and community-levels, but without individual HIV/ART status. Model 3 examined the effect of all individual-level predictors on TB, but without community predictors. Model 4 was the full model that considered all the explanatory variables at individual and community levels. Model fit was assessed based on Akaike’s Information Criterion (AIC)^48^, where the lower value indicates a better fit. A model comparison was conducted using a post-estimate likelihood-ratio (LR) test for the nested models. For each model, variance and the intra-class correlation coefficients (ICC), which explains the proportion of total variance at individual- and community-level, were computed.

## Results

**Figure 1:**
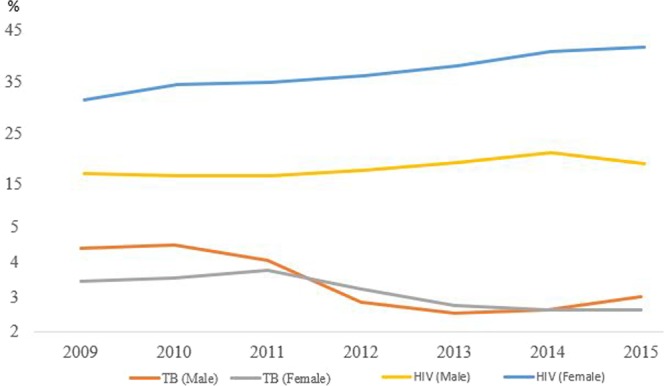
Estimated HIV prevalence for males (15–49) and females (15–54) in the study surveillance area. Percentage (y-axis) depicts recently-diagnosed TB within the previous 12 months. Horizontal number indicates year.

The sample cohort consisted of 41,812 individuals ages 15 years and above and consisted of females (n = 25,218) and male (n = 16,594) who responded to the TB question from 2009 to 2015. The number of individuals with recently-diagnosed TB annually from 2009 to 2015 is shown in Fig. 1. The proportion of respondents with recently-diagnosed TB (within the previous 12 months) was 3.23% from 2009 to 2015, where the estimate ranged from 2.64% (lowest in 2014) to 3.86% (highest in 2010). There was a gradual decline in recently-diagnosed TB after 2011 until 2014, as indicated in Fig. 1.

### Space-time clustering of TB

**Figure 2:**
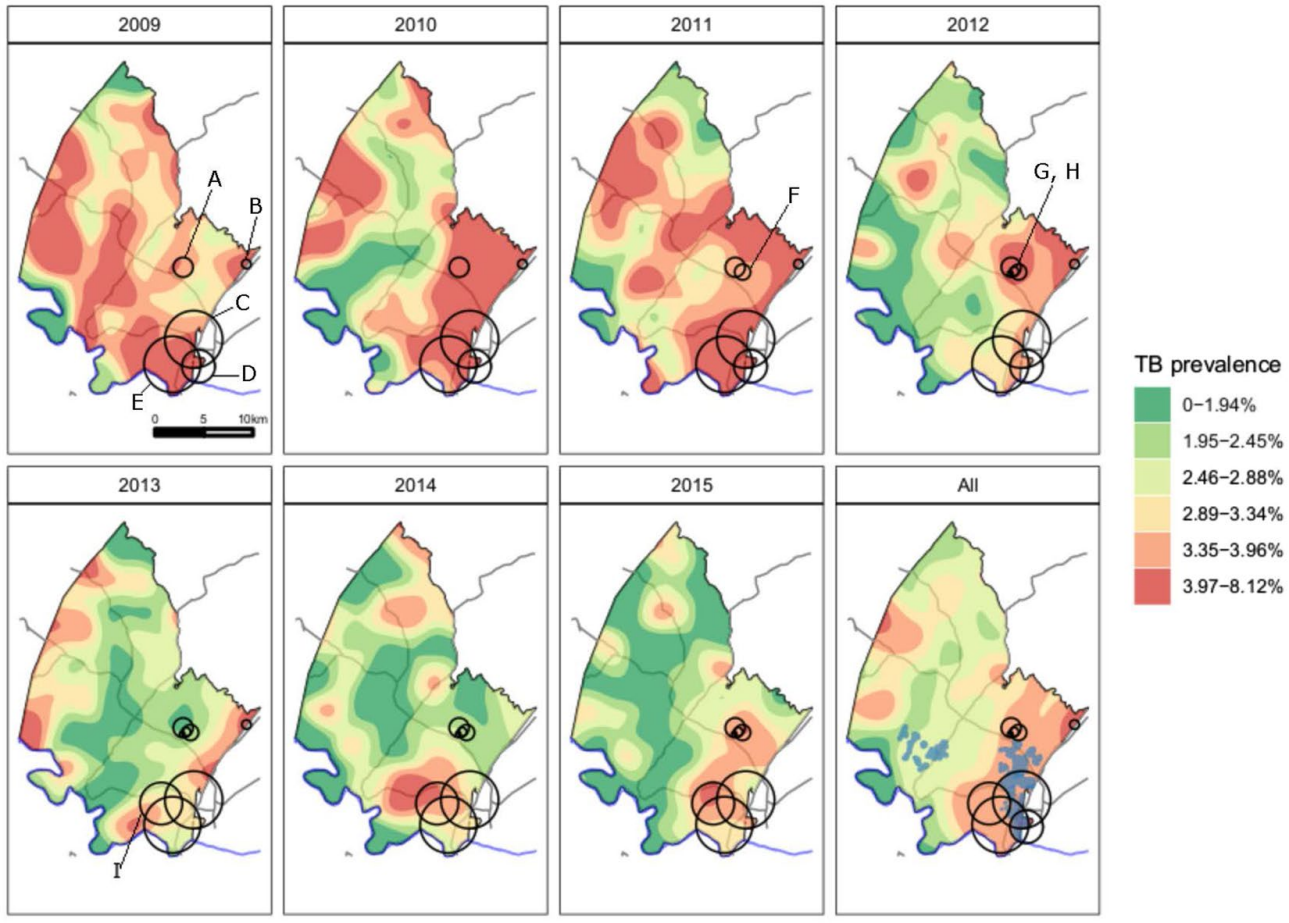
The study area with high-risk, overlapping space-time recently-diagnosed TB clusters (p < 0.05) identified by the Kulldorff statistic in peri-urban communities near the National Road (in grey color)^41^. The National Road continues along the eastern boundary of the surveillance area towards Mozambique. “All” panel shows locations of TB clusters through the entire study period, overlaid on the average prevalence of recently-diagnosed TB. Blue shaded areas show locations of previously identified HIV clusters^43^. Cluster relative risks: A, 2.1; B, 4.4; C, 1.3; D, 1.6; E, 1.3; F, 3.3, G, 6.2, H, 10.1; I, 1.9.

There were nine high-risk space-time clusters of recently-diagnosed TB (p < 0.05, Fig. 2), with description being provided in Table 1. The four largest clusters (denoted E, C, D, I) were in overlapping areas in the south east of the study area, a peri-urban community near the national highway, three of which (E, C, D) persisted throughout the study period. The areas of TB clustering in the south east of the study area also overlapped with previously identified HIV clusters^43^. There was also a smaller area of sustained clustering further north in the study area (A), with overlapping clusters (F, G, H) that were detected for shorter time periods (Table 1).

**Table 1:** Description of the space-time clusters of recently-diagnosed TB.

Cluster	Radius (Km)	Start Year	End Year	LLR	P-value	Observed	Expected	Relative Risk	Mean prevalence of recently-diagnosed TB (%)
A	1.00	2009	2015	19.66	<0.01	93	45.21	2.09	3.61
B	0.47	2009	2013	17.14	0.02	24	5.43	4.44	5.14
C	2.94	2009	2015	24.27	<0.01	856	690.06	1.33	3.78
D	1.69	2009	2012	25.48	<0.01	275	175.87	1.62	4.70
E	2.88	2009	2015	19.00	<0.01	900	749.61	1.28	3.73
F	0.80	2011	2015	22.27	<0.01	46	14.27	3.26	3.30
G	0.46	2012	2015	28.34	<0.01	29	4.74	6.16	3.23
H	0.27	2012	2015	19.79	<0.01	14	1.39	10.14	3.17
I	2.16	2013	2015	20.26	<0.01	120	63.65	1.92	3.54

LLR stands for log likelihood ratios. TB stands for tuberculosis.

### Evaluating the impact of ART scale-up in TB outcome

The results of the bivariate analysis (Table 2) of individual risk predictors suggested that while communities with higher HIV prevalence were associated with higher (odds of) recently-diagnosed TB, individuals residing in communities with greater ART coverage were associated with lower recently-diagnosed TB. However, older individuals living with HIV who were also on ART from lower socio-economic households were most likely to report recently-diagnosed TB.

**Table 2:** Mixed-effects models assessing the relationship between individual/household/community factors on recently-diagnosed TB.

Level	Variable	Category	Bivariate Analyses		Null model		Model 1		Model 2		Model 3		Model 4	
			OR	SE	aOR	SE	aOR	SE	aOR	SE	aOR	SE	aOR	SE
Level - Individual:	Age category: [Male 15-24]	Male 25-29	5.69***	1.41			5.68***	1.39	5.15***	1.28	3.30*	1.72	3.24*	1.68
		Male 30-34	20.94***	4.94			20.91***	4.90	19.86***	4.71	4.74**	2.33	4.59**	2.26
		Male 35-39	43.38***	10.11			45.23***	10.66	40.79***	9.75	7.17***	3.46	6.97***	3.37
		Male 40-44	48.28***	11.96			58.46***	14.76	54.28***	13.81	9.28***	4.62	9.16***	4.56
		Male 45-49	47.83***	11.93			65.23***	16.70	61.81***	15.99	10.56***	5.45	10.07***	5.20
		Male 50-54	41.37***	10.61			59.77***	15.85	57.28***	15.34	7.38***	3.94	7.16***	3.83
		Male 55-59	31.03***	8.69			52.42***	15.13	49.13***	14.45	14.13***	8.34	14.07***	8.32
		Male 60+	12.08***	2.77			22.80***	5.45	21.99***	5.31	3.08	2.00	3.07	1.99
		Female 15-24	1.75**	0.31			1.79**	0.32	1.73**	0.32	1.01	0.46	0.98	0.45
		Female 25-29	8.33***	1.63			8.49***	1.64	8.35***	1.63	1.91	0.86	1.86	0.84
		Female 30-34	15.86***	3.15			17.05***	3.36	17.00***	3.39	3.23**	1.45	3.12*	1.40
		Female 35-39	18.11***	3.65			21.83***	4.39	20.56***	4.19	3.47**	1.57	3.40**	1.54
		Female 40-44	12.78***	2.68			17.42***	3.68	16.26***	3.49	3.28*	1.52	3.21*	1.48
		Female 45-49	13.70***	2.79			19.21***	3.99	18.61***	3.91	5.12***	2.37	5.01***	2.32
		Female 50-54	9.91***	2.07			14.39***	3.07	13.79***	2.97	3.91**	1.85	3.80**	1.81
		Female 55-59	6.97***	1.60			10.01***	2.36	10.04***	2.38	4.33**	2.16	4.21**	2.11
		Female 60+	4.12***	0.77			6.17***	1.28	6.00***	1.26	3.71*	1.93	3.69*	1.92
	Marital Status: [Single]	Divorced/Separated	1.05	0.12			0.72*	0.10	0.73*	0.10	0.66*	0.13	0.65*	0.13
		Marriage - Monogamous	1.03	0.11			0.39***	0.04	0.42***	0.05	0.58**	0.10	0.57**	0.10
		Marriage - Polygamous	0.75	0.20			0.37***	0.10	0.39***	0.10	0.62	0.30	0.61	0.30
	HIV status: [Negative]	Positive	9.87***	0.91							3.59	2.95	3.83	3.18
		Indeterminate/Not Applicable	1.56***	0.13							2.02	1.66	2.12	1.76
	ART initiation: [HIV+ and ART not initiated]	HIV+ and ART initiated	3.87***	0.40							4.05***	0.48	4.23***	0.51
	Year [2009-2010]	Year 2010 and before	1.12	0.09			1.09	0.09	1.02	0.09	1.26	0.16	1.24	0.16
		Year 2011 and on	0.94	0.07			0.91	0.07	0.91	0.09	0.96	0.11	1.29	0.18
Level - Household:	Household income quintile [Lowest 20%]	Lower 20%	0.95	0.10					0.82	0.09	0.89	0.13	0.93	0.13
		Middle 20%	0.79*	0.08					0.85	0.09	0.77	0.11	0.8	0.12
		Higher 20%	0.77*	0.08					0.67***	0.07	0.84	0.12	0.87	0.13
		Top 20%	0.56***	0.06							0.72*	0.11	0.73*	0.11
Level - Community:	ART coverage	ART %	0.99***	<0.01					1.00	<0.01			0.98***	0.01
	HIV prevalence	HIV %	1.03***	<0.01					1.03***	0.01			0.98	0.01
	Typography [Peri-urban]	Rural	0.80**	0.06					0.98	0.10			0.88	0.12
		Urban	1.03	0.13					0.78	0.14			1.38	0.30
	Variance components:	Individual			5.58 (0.43)		4.45 (0.40)		4.45 (0.40)		2.83 (0.55)		2.85 (0.55)	
		Community			1.31 (0.21)		1.08 (0.21)		0.99 (0.21)		0.69 (0.37)		0.67 (0.37)	
	Intraclass correlation:	Individual			67.68%		62.71%		62.23%		51.66%		51.71%	
		Community			12.91%		12.26%		11.33%		10.12%		9.84%	
	Model fit:	AIC			15793.26		14765.36		14395.66		6346.52		6341.00	

Reference category in bracket. *p < 0.05, **p < 0.01, ***p < 0.001. ICC stands for intraclass correlation. TB stands for tuberculosis. HIV stands for human immunodeficiency viruses. ART stands for antiretroviral therapy. Bivariate mixed-effect logistic models are based on level specification unique to each variable. The year in the model was separated before and after 2011 due to change in the adult treatment eligibility thresholds in August 2011.

The results from the five multivariate multilevel models are presented in Table 2. The intraclass correlation (ICC) from model 0 indicated that approximately 13% of the variation in recently-diagnosed TB outcome can be attributed to differences between communities. Model 1 indicated that older age and male gender were risk factors for recently-diagnosed TB, but being married or separated (compared to being single) was a protective factor against the outcome. As mentioned earlier, the main individual-level outcome was TB, based on self-report, and recently-diagnosed within the previous 12 months between 2009–2015.

Model 2, which added community and household explanatory variables to Model 1, indicated that higher HIV prevalence was significantly associated with more recently-diagnosed TB (aOR = 1.03, 95% CI: 1.01–1.04), with greater household income being a protective factor against the outcome (aOR = 0.67, 95% CI: 0.54–0.84). The significance of the findings from Model 1 (gender, age, marital status) remained the same in Model 2. Model 3, which added HIV status and ART initiation to Model 1, suggested that those who were HIV + that initiated ART were significantly more likely to report recently-diagnosed TB (aOR = 4.05, 95% CI: 3.21–5.11).

**Figure 3:**
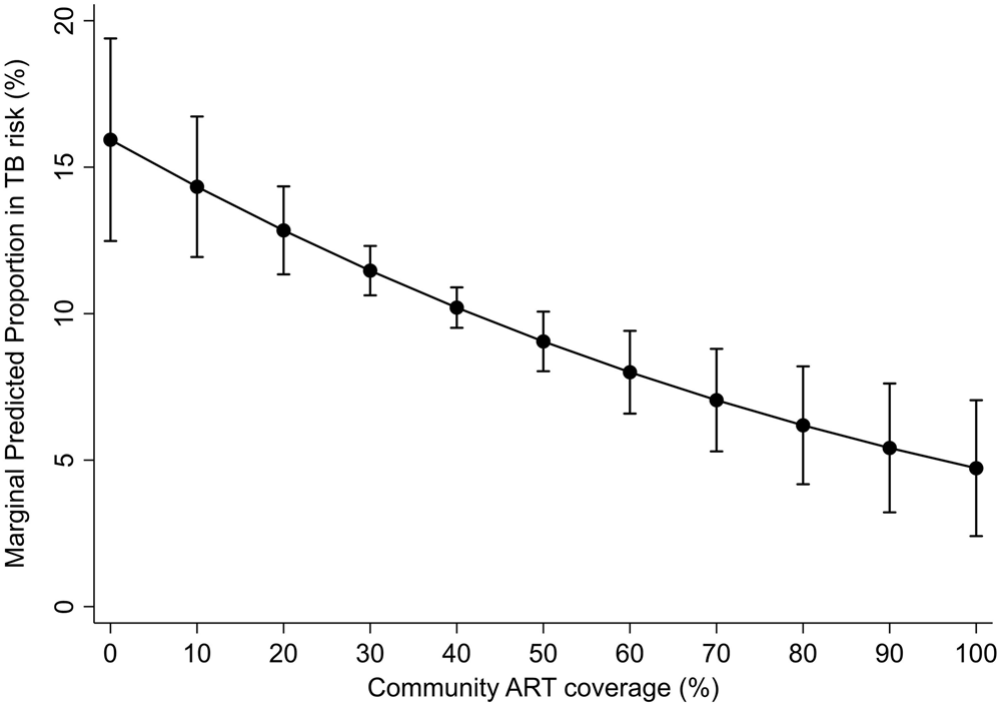
Margin plot of community ART coverage and recently-diagnosed TB

Model 4, a full model that controlled for all explanatory variables, indicated that higher community ART-coverage was significantly associated with lower recently-diagnosed TB (aOR = 0.98, 95% CI: 0.97–0.99) over and above the individual effect (when the individual ART status is controlled for in Model 3). The association between community-ART coverage and recently-diagnosed TB, based on Model 4 post-estimation margins, is plotted in Fig. 3. The significance of the findings from Model 3 (gender, age, marital status, and ART initiation) remained the same in Model 4. Among the four models, Model 4 had the most appropriate fit, based on the lowest AIC. Model comparison (Model 3 nested in Model 4), using likelihood-ratio test, indicated that adding community-level explanatory variables to Model 3 results in a statistically significant improvement in model fit (LR χ^2^ = 13.52, df = 4, *p* < 0.01). The ICC from Model 4 indicated that 10% of the variation in recently-diagnosed TB outcome can be attributed to community-level factors.

## Discussion

South Africa continues to experience considerable mortality and morbidity from the combined epidemics of TB and HIV^49^. As momentum grows to end the two diseases, we need a better understanding of the epidemics to inform locally targeted responses^50^. In this analysis of data from a well-characterized rural South African population with high HIV prevalence and TB incidence, we have demonstrated considerable spatial heterogeneity in people with recently-diagnosed TB, and have shown that every percentage increase in ART coverage was associated with a 2% decrease in the odds of recently-diagnosed TB (aOR = 0.98, 95% CI:0.97–0.99). Our study finding is consistent with other investigations that found spatial heterogeneity in people with drug-resistant TB in the region^51^, including in our surveillance area^52^. These results give support to the assumption that increasing ART coverage is contributing to the local control of TB.

This study was conducted in a population with an extremely high TB case notification rate, where the epidemic is fueled by the high prevalence of HIV. Over the study period, approximately 3% of community members 15 years or older reported a TB diagnosis in the previous year. This is higher than the nationally representative South African National Income Dynamics Study (SA-NIDS)^53^, which reported a prevalence of self-reported TB of 1.6% in 2008 and 0.6% in 2012^54^. Our spatial analysis uncovered local areas of sustained high prevalence of recently-diagnosed TB in the township and surrounding peri-urban communities on the edge of the study area, near the national highway. We have previously demonstrated that this is an area of particularly high HIV prevalence and incidence, which also has a high risk of rifampicin-resistant TB (RR-TB)^40,43,52^. This suggests that our previous finding of a high risk of RR-TB in this area was largely related to a high risk of TB in general, and not to specific clustering of drug resistance^52^.

To define recently-diagnosed TB, we relied on participant self-report of TB collected as part of a comprehensive population-based health surveillance programme. However, we were unable to validate this measure against laboratory or TB programme records. Validity of the self-report measure could be affected by misunderstanding the survey question or medical terminology, misreporting TB by participants, or fieldworker error in questioning or documenting the responses. It is possible that fieldworker error could be unevenly distributed across the study area and could therefore bias the estimates of prevalent TB in certain areas. However, it should be noted that the entire cadre of study fieldworkers visit an area at a particular time, and they complete the whole area together. There are no areas allocated to a single fieldworker, and the pattern of fieldwork is unlikely to introduce a substantial bias.

To our knowledge, no large-scale population studies of TB distribution have assessed the validity of self-reported TB as a health outcome. However, the spatial distribution of TB we have described in this study is consistent with independent work focusing on the spatial distribution of drug resistant TB cases from the same district^52^ as well as the spatial distribution of population-based HIV prevalence^40^. Thus, although we cannot completely rule out the existence of such a bias, it is highly unlikely that this could explain our findings. Other groups have also reported problems with the validity of self-reported HIV status and antiretroviral use in population-based surveys in South Africa and other African countries, usually driven by under-reporting^55–57^. If stigma or social desirability bias drives that under-reporting, it is plausible that it might also apply to survey questions about TB. This is also an argument in our study, that our self-reported TB (i.e. recently-diagnosed TB within the previous 12 months) is high due to errors in time perception. While recall bias is a possibility, a previous study on the validity of self-reported data has shown that under-reporting remained the highest in the 12 months period (compared to 30, 91 or 183 recall periods) in an aggregated health measure^58^. Nonetheless, data from the District Health Barometers^59^ suggests that the TB case notification rate is consistently higher than the national average in our study area. In 2008, immediately prior to the study period, TB case notification rate in Hlabisa sub-district was 1700 per 100 000, considerably above the national average^60^. We would therefore expect our TB estimates to be higher than the figure from other nationally representative survey, such as SA-NIDS. The other inherent limitation of trying to understand spatial distribution of TB disease from self-reported TB data is that undiagnosed TB cases will not be included. The prevalence of undiagnosed TB may be geographically heterogeneous, especially if it is affected by access to health care facilities or by differential case detection performance at facilities.

Interpreting our findings relating to the impact of community ART coverage on active TB (i.e. recently-diagnosed TB) may be subject to other limitations. Participation levels were relatively modest (below 50%) for the general health survey, and lower for men than women, although over two-thirds participated at least once in each five-year period in the study area^61^, which could contribute to bias. However, the temporal consistency of the spatial distribution of recently-diagnosed TB over the study period make this unlikely. Despite modest participation for the general health survey, approximately 90% of our study participants responded to the TB question, and we identified remarkably consistent spatial pattern over time. This suggests limited impact due to any participation rates and non-response bias.

Secondly, our definition of ART coverage was based on the proportion of all HIV-infected individuals receiving ART in a particular community, based on linkage to facility-based data. The estimates of ART coverage may therefore be an over-estimation, which does not account for people disengaging from care^62^. If disengagement from care is not distributed equally across the study area, this could lead to errors in defining the association between ART coverage and TB. However, such error would bias the result towards the null hypothesis of no ART impact.

Thirdly, there is the possibility that clinicians, mistakenly, may be less inclined to diagnose TB in individuals on ART. Some of these points may explain the lower TB reported during the recent scale-up of community ART and the introduction of Xpert MTB/RIF into our study area and warrants confirmation of our findings using bacteriologically proven measures of TB in future investigations. In our current study, we did not have data on the proportion of bacteriologically confirmed TB diagnoses. Xpert was only introduced in our study area in mid-2013, and so the impact, if any, on reducing empirical TB treatment would have been limited to the last few months of the study period. Lastly, data on isoniazid preventive therapy (IPT) was not available, and we could therefore not establish to what extent this may have contributed to the population-level reduction in TB. Whilst IPT has been recommended for persons living with HIV since the start of ART roll-out in South Africa in 2004, implementation has been relatively weak^63^, and thus unlikely to have had any major bearing on the findings. Even by 2015, just after the end of our study period, IPT coverage among PLHIV newly enrolled in care nationally was only 38%^64^. Furthermore, lack of data on levels of IPT completion makes it even more difficult to estimate its effect at a population level. In conclusion, we found clear evidence of substantial space-time clustering of TB in a predominantly rural community in South Africa with a severe TB epidemic driven by HIV. Furthermore, we found a significant association between increased community ART coverage and lowering of recently-diagnosed TB at a community level. Whilst the exact mechanism behind this reduction remains unclear, this provides support for the development and evaluation of precision public health strategies to end TB and HIV in sub-Sahara Africa.

## Data Availability

Data are available upon request from the Africa Health Research Institute (from https://data.africacentre.ac.za)
